# Improvements in sleep‐disordered breathing during acclimatization to 3800 m and the impact on cognitive function

**DOI:** 10.14814/phy2.14827

**Published:** 2021-05-15

**Authors:** Shyleen Frost, Jeremy E. Orr, Britney Oeung, Nikhil Puvvula, Kathy Pham, Rebbecca Brena, Pamela DeYoung, Sonia Jain, Shelly Sun, Atul Malhotra, Erica C. Heinrich

**Affiliations:** ^1^ Division of Biomedical Sciences School of Medicine University of California Riverside CA USA; ^2^ Division of Pulmonary, Critical Care, Sleep Medicine and Physiology School of Medicine University of California San Diego CA USA; ^3^ Biostatistics Research Center, Herbert Wertheim School of Public Health and Human Longevity Science University of California San Diego CA USA

**Keywords:** cognition, high altitude, sleep, sleep‐disordered breathing

## Abstract

Sojourners to high altitude often experience poor sleep quality due to sleep‐disordered breathing. Additionally, multiple aspects of cognitive function are impaired at high altitude. However, the impact of acclimatization on sleep‐disordered breathing and whether poor sleep is a major contributor to cognitive impairments at high altitude remains uncertain. We conducted nocturnal actigraphy and polygraphy, as well as daytime cognitive function tests, in 15 participants (33% women) at sea level and over 3 days of partial acclimatization to high altitude (3800 m). Our goal was to determine if sleep‐disordered breathing improved over time and if sleep‐disordered breathing was associated with cognitive function. The apnea–hypopnea index and oxygen desaturation index increased on night 1 (adj. *p* = 0.026 and adj. *p* = 0.026, respectively), but both improved over the subsequent 2 nights. These measures were matched by poorer self‐reported sleep quality on the Stanford Sleepiness Scale and PROMIS questionnaires following 1 night at high altitude (adj. *p* = 0.027 and adj. *p* = 0.022, respectively). The reaction time on the psychomotor vigilance task was slower at high altitude and did not improve (SL: 199 ± 27, ALT1: 224 ± 33, ALT2: 216 ± 41, ALT3: 212 ± 27 ms). The reaction times on the balloon analog risk task decreased at high altitude (SL: 474 ± 235, ALT1: 375 ± 159, ALT2: 291 ± 102, ALT3: 267 ± 90 ms), perhaps indicating increased risk‐taking behavior. Finally, multiple cognitive function measures were associated with sleep‐disordered breathing and measures of subjective sleep quality, rather than low daytime arterial oxygen saturation. These data indicate that sleep‐disordered breathing at moderately high altitude improves with partial acclimatization and that some aspects of cognitive performance in unacclimatized sojourners may be impacted by poor sleep rather than hypoxemia alone.

## Graphical abstract

Sleep‐disordered breathing and impaired cognitive performance are common at high altitudes but the relationship between the two factors remains unclear. We found that sleep‐disordered breathing and subjective sleep quality improve over 3 days of acclimatization to 3800 m elevation. However, despite these improvements, daytime sustained attention and reaction time remained impaired.

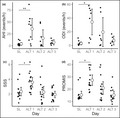

## 1 INTRODUCTION

High‐altitude exposure can produce several physiological and neurocognitive impairments due to reduced oxygen availability and other environmental stressors such as low humidity or temperature. Varying degrees of cognitive impairment upon acute high‐altitude exposure have been described, including impairments in complex reaction time (Kramer et al., 1993; Mackintosh et al., 1988), psychomotor performance (Bolmont et al., 2000), and memory (Kramer et al., 1993). The severity of these impairments increases with elevation (Virués‐Ortega et al., 2004; Yan, 2014). Although different test batteries, ascent profiles, and individual variation in effort and the degree of hypoxemia lead to inconsistent results for some cognitive domains, particularly in mild and moderate hypoxic conditions (Petrassi et al., 2012), complex reaction time is consistently impaired across numerous studies (Virués‐Ortega et al., 2004). While these effects have been well documented, multiple factors may contribute to these changes in cognitive function and the precise mechanisms warrant further investigation.

Possible contributors to cognitive impairment during acute high‐altitude exposure are low arterial oxygen pressure, Acute Mountain Sickness (AMS) symptoms, and poor sleep quality. Brief simulated altitude experiments show that acute changes in inspired oxygen can impact vision, reaction time, and working memory (Malle et al., 2016; Phillips et al., 2015), indicating that hypoxemia itself contributes to poor cognitive function. There is also evidence that AMS is associated with poor cognitive performance (Issa et al., 2016; Regard et al., 1991; Shukitt‐Hale et al., 1991); however, cognitive impairments are also seen in the absence of AMS symptoms. A recent study with a large sample shows no significant correlation between cognitive performance and AMS scores (Phillips et al., 2017).

Sleep disruption impacts daytime cognitive performance in healthy individuals as well as those with conditions such as obstructive sleep apnea (Latshang et al., 2013). Obstructive sleep apnea is linked to deficits in attention, memory, executive function, and psychomotor function (Olaithe et al., 2018). Sleep‐disordered breathing (SDB) is common following ascent to high altitude (Nussbaumer‐Ochsner et al., 2012). SDB may occur when high hypoxic ventilatory drive causes periods of hyperventilation which produce subsequent hypocapnia‐driven hypopneas or apneas (Ainslie et al., 2013). These waxing and waning breathing patterns produce important intermittent desaturations and arousals that decrease sleep quality and can contribute to poor daytime cognitive function.

Studies examining the effects of acclimatization on sleep have produced varying results depending on the altitude, ascent profile, and exposure time (Ainslie et al., 2013; Andrews et al., 2012; Bloch et al., 2010; Burgess et al., 2013; Horiuchi et al., 2017; Insalaco et al., 2012; Nussbaumer‐Ochsner et al., 2012; Tseng et al., 2015; White et al., 1987). In general, SDB seems to persist or increase in severity with acclimatization at very high altitudes (>4500 m) (Andrews et al., 2012; Burgess et al., 2013; Insalaco et al., 2012; Nussbaumer‐Ochsner et al., 2012), whereas at high altitudes (<4500 m) periodic breathing may persist (Horiuchi et al., 2017) or improve (Tseng et al., 2015; Weil, 2004; White et al., 1987; Wickramasinghe & Anholm, 1999) with acclimatization. Fewer studies have examined the impact of acclimatization on cognitive function although a recent study by Pun et al., (2018) found improvements in selective and sustained attention with 6 days of acclimatization. Whether neurocognitive impairments at high altitude are driven by poor sleep quality or SDB versus hypoxemia or AMS severity remains to be determined. The aim of this study was to measure changes in cognitive performance during acclimatization to high altitude and determine whether a cognitive performance was predicted by AMS scores, resting arterial oxygen saturation, and/or sleep‐disordered breathing. We hypothesized that poor sleep is a major contributor to impaired cognition at high altitude.

## 2 METHODS

### 2.1 Ethical approval

This study was approved by the University of California, Riverside Clinical Institutional Review Board (HS 19–076). All participants were informed of the study's purpose and risks. Participants provided written informed consent in their native language (English). The work was conducted in accordance with the *Declaration of Helsinki*, except for registration in a database.

### 2.2 Participants

A total of 15 participants were recruited (10 men and 5 women). All participants were healthy individuals between 19 and 32 years old and had no history of cardiovascular or pulmonary disease. The average age for males was 24.9 (4.3) years with an average BMI of 26.7 (5.4). For females, the average age was 26.4 (5.1) with an average BMI of 28.4 (6.9). Exclusion criteria included smoking (cigarettes, e‐cigarettes, marijuana), pregnancy, travel to altitudes greater than 2500 m within one month prior to the first test measurement, or use of anti‐inflammatory medications (i.e., ibuprofen) that can interfere with acclimatization to high altitude (Basaran et al., 2016).

### 2.3 Experimental design

Participants completed cognitive function testing and sleep measures at sea level and over 3 days at high altitude. Sea‐level measures were completed at the University of California, Riverside (340 m elevation) and high‐altitude measures were taken at Barcroft Station within the White Mountain Research Center (3800 m). Participants were driven to Barcroft Station and ascended from 340 m to 1216 m over 4 hours, then from 1216 m to 3800 m in 2 h.

Physiological measures and questionnaires were collected each morning. After these measures, participants were permitted to eat a light breakfast prior to completing cognitive function tests but did not consume caffeine until completing the cognitive test battery. During the study, participants were asked to abstain from taking nonsteroidal anti‐inflammatory drugs (NSAIDs) or acetazolamide (Basaran et al., 2016).

### 2.4 Measurements

#### 2.4.1 Physiological measurements and questionnaires

Heart rate (HR), blood pressure (BP), and resting daytime oxygen saturation (SpO_2_) were measured each morning at sea level and high altitude. BP measurements were collected with a manual sphygmomanometer. HR and daytime SpO_2_ were measured with a pulse oximeter (Nellcor N600, Medtronic) using a fingertip probe. Participant sat upright in a chair with their feet on the ground and legs uncrossed and were asked to rest for 2–3 minutes until SpO_2_ stabilized.

Participants verbally completed the Lake Louise AMS Score questionnaire each morning (Ulrich et al., 2018). Participants completed the *Pittsburgh Sleep Quality* *Index* (PSQI) during their baseline sea‐level visit (Smyth, 2011). The PSQI was used to determine the participant's baseline sleep quality and patterns. They also completed the Stanford Sleepiness Scale (SSS) and a modified version of the short form 8‐item PROMIS Sleep Disturbance questionnaire each morning (substituting the timeframe for each question from “past 7 days” to “past 1 day”). PROMIS T‐scores were calculated based on the 2013 sleep disturbance 8b short form conversion table.

#### 2.4.2 Cognitive function

Participants completed a 30‐min cognitive function test battery (*Cognition* by Joggle Research) once at sea level and once each morning over 3 days at 3800 m elevation. The test battery consisted of eight different tasks that used different measures (reaction time, accuracy, number of correct responses) to determine performance in each cognitive domain (Table 1). A detailed description of each test can be found in the supplemental material or at the Joggle Research website (https://admin.joggleresearch.com/Home/Tasks). The cognitive test battery was taken on a 12.9‐inch iPad Pro (Apple, Inc.). Participants completed the tests in a separate, quiet room to eliminate any sources of distraction, and were seated in an upright position with the iPad placed on a desk in front of them. Before each test, instructions were presented on the screen to eliminate any variance introduced by a researcher explaining tests to participants. In addition to the instructions, some assessments had a practice session which familiarized participants with the test prior to completing the experimental session. To prevent learning effects, participants were provided with a novel array of test permutations during each test session. Additionally, half (n = 8) of the participant group was assigned to complete sea‐level cognitive function testing before the ascent to high altitude and a half (n = 7) completed baseline tests at least 2 days after descent to sea level to verify that performance at sea level compared to day 1 at altitude was not associated with the order in which tests were taken.

**TABLE 1:** *Cognition* test battery description

Test name	Abbreviation	Cognitive domain
Psychomotor vigilance task	PVT	Vigilant attention
Balloon analog risk task	BART	Risk decision making
Digit symbol substitution task	DSST	Complex scanning and visual tracking
Line orientation task	LOT	Spatial orientation
NBack	NBACK	Working memory
Visual object learning task	VOLT	Visual learning and spatial working memory
Abstract matching	AM	Abstraction
Motor praxis task	MPT	Sensory motor speed

#### 2.4.3 Sleep studies

Actigraphy and polygraphy were used to measure SDB. Participants were assigned an Actiwatch (Philips Respironics) to wear at sea level for 1–3 days and throughout the duration of their 3‐day stay at White Mountain Research Center (Barcroft Station, 3800 m). Participants were also instrumented with respiratory polygraphy (Apnealink Air, ResMed) for 1 night at sea level and each night while at altitude. The Apnealink Air was chosen because it is designed to allow participants to activate the device on their own after training for simple at‐home testing at sea level. These methods were used to quantify SDB via apnea–hypopnea index (AHI), oxygen desaturation index (ODI), minutes of wakefulness after sleep onset (WASO), sleep efficiency, and nocturnal pulse oximetry. In the morning, SSS and PROMIS questionnaires assessing their subjective sleep and health were obtained as described above. The actigraphy and polygraphy data were scored blindly by a registered polysomnographic technologist using American Academy of Sleep Medicine criteria for scoring and Chicago criteria for events via Philips Actiware 6 software and Airview, respectively. Due to equipment limitations and subject adherence, complete sleep studies were obtained from ten individuals at sea level, nine on night 1 at high altitude, eight on night 2, and six on night 3.

### 2.5 Statistical analysis

All statistical analyses were performed in R version 3.6.1 (R Inc.). Wilcoxon signed‐rank tests were used to determine significant changes in sleep‐disordered breathing and cognitive performance on each day at high altitudes (ALT1, ALT2, and ALT 3) compared to sea‐level (SL) performance. Pairwise Spearman's correlations were used to determine if cognitive performance scores on the first day at high altitude were associated with physiological variables such as AMS scores and daytime SpO_2_, or sleep measures including SSS and PROMIS questionnaires, AHI, hypopnea index, apnea index, central apnea index, and ODI. The Benjamini–Hochberg procedure was used to adjust for multiple comparisons with a false discovery rate of 0.05. Raw and adjusted p values are reported. Data are presented throughout the manuscript as mean (standard deviation). Asterisks indicate significant differences at *p *< 0.05 (*), *p *< 0.01 (**), *p *< 0.001 (***), or *p*<0.0001(****). Cognitive function data were missing for one participant who completed cognitive function tests after returning to sea level due to an unknown software error and therefore this subject was excluded from the cognition analysis.

## 3 RESULTS

### 3.1 High‐altitude effects on physiological measures

Table 2 provides an overview of the physiological measures taken each morning. There was no significant change in systolic blood pressure during high‐altitude exposure. Diastolic blood pressure was slightly higher than sea‐level values on day 1 and 3 at high altitude, but this difference was not significant after correcting for multiple comparisons. Resting heart rate increased and remained higher throughout the stay at high altitude. Resting SpO_2_ decreased at high altitude and remained lower than sea‐level values throughout the stay at high altitude. AMS Scores were significantly higher on the first two mornings at high altitude but returned to baseline levels by day 3.

**TABLE 2:** Physiological measures collected each morning at sea level (SL) and high altitude (ALT) days 1–3 (n = 15)

Measure	SL	ALT 1	*p*	ALT 2	*p*	ALT 3	*p*
P_systolic_	129 (7.5)	125 (12.2)	0.315	126 (11.7)	0.53	126 (12.7)	0.315
P_diastolic_	79 (10.1)	83 (8.8)	0.043	83 (7.3)	0.084	85 (6.9)	0.033
Resting HR	78 (8.1)	88 (13.2)	<0.001*	90 (12.1)	0.004*	96 (12.8)	<0.001*
Resting daytime SpO_2_	95 (1.6)	85 (4.4)	<0.001*	84 (2.6)	<0.001*	86 (2.6)	<0.001*
AMS Score	0.2 (0.4)	3.1 (1.8)	<0.001*	2.3 (2.0)	0.004*	0.7 (1.2)	0.222

Data are presented as mean (standard deviation). Raw *p* values are provided and asterisks indicate significant differences from SL after B‐H adjustment for multiple comparisons. Units: P (mm Hg), HR (bpm), SpO_2_ (%).

### 3.2 High‐altitude effects on cognitive performance

**FIGURE 1:**
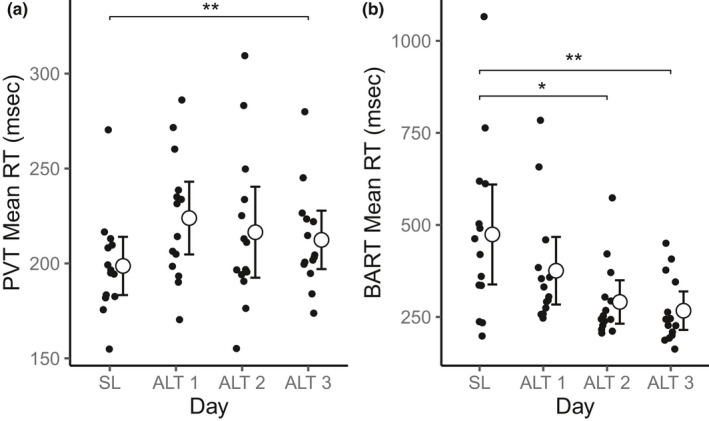
Cognitive function tests demonstrating significant effects of high altitude on performance. Plots demonstrate significant differences in reacting time on the PVT (A) and BART (B) tests across days. Asterisks indicate significant differences from SL *p *< 0.05 (*), *p *< 0.01 (**), *p *< 0.001 (***), or *p *< 0.0001(****) after correcting for multiple comparisons

Table 3 provides an overview of the effects of high altitude on test performance. Mean reaction time on the PVT was slower each day at high altitude compared to sea level and remained significantly slower on day 3 (Figure 1a). There was no difference in the number of lapses or false starts on the PVT. Mean reaction time per click on the BART was lower at high altitude on days 2 and 3 compared to sea level (Figure 1b), but there was no effect on the number of pumps per balloon. Altitude did not significantly impact performance on any other cognitive function test after correcting for multiple comparisons.

**TABLE 3:** Effects of day on cognitive test performance measures

Test	Measure	SL	ALT 1	*p*	ALT 2	*p*	ALT 3	*p*
PVT	Mean RT	189 (25)	224 (33)	0.03	217 (42)	0.02	212 (27)	<0.001*
	Lapses	1.0 (1.3)	1.9 (2.7)	0.16	1.9 (2.4)	0.085	1.4 (2.0)	0.305
	False starts	2.6 (4.3)	1.8 (1.5)	0.905	1.0 (0.7)	0.12	0.6 (0.8)	0.057
BART	Mean RT	474 (235)	376 (159)	0.078	297 (101)	0.002*	267 (91)	<0.001*
	Pumps	3.9 (1.0)	4.1 (1.1)	0.67	3.7 (1.4)	0.433	3.7 (0.9)	0.502
DSST	Mean RT	851 (96)	876 (65)	0.068	854 (66)	0.542	825 (58)	0.326
	CR	94.3 (9.2)	91.7 (6.2)	0.062	93.9 (6.4)	0.656	96.8 (6.2)	0.324
LOT	Mean RT	6076 (1831)	5173 (988)	0.025	5051 (1235)	0.03	4928 (1248)	0.068
	CR	13.9 (4.0)	13.4 (3.7)	0.723	13.5 (2.4)	0.672	14.3 (3.3)	0.888
NBACK	Mean RT	567 (66)	568 (64)	0.808	514 (58)	0.007	541 (86)	0.153
	CR	53.5 (4.6)	52.2 (5.2)	0.223	53.6 (4.8)	1	53.8 (3.8)	0.806
VOLT	Mean RT	1969 (809)	1918 (603)	0.855	1748 (616)	0.542	1590 (568)	0.058
	CR	17.3 (2.0)	16.1 (2.1)	0.203	17.1 (1.7)	0.718	17.6 (2.2)	0.581
AM	Mean RT	1928 (968)	2013 (789)	0.903	1704 (632)	0.296	1721 (522)	0.358
	CR	19.0 (4.2)	17.6 (3.3)	0.35	18.5 (3.7)	0.688	19.4 (3.3)	0.844
MPT	Mean RT	400 (47)	401 (24)	0.761	410 (53)	0.432	397 (57)	0.715

Data are presented as mean (standard deviation). Reaction times are provided in ms. Raw *p* values are provided and asterisks indicate significant differences from SL after B‐H correction for multiple comparisons. RT, reaction time; CR, correct responses.

### 3.3 High‐altitude effects on SDB and subjective sleep quality

**FIGURE 2:**
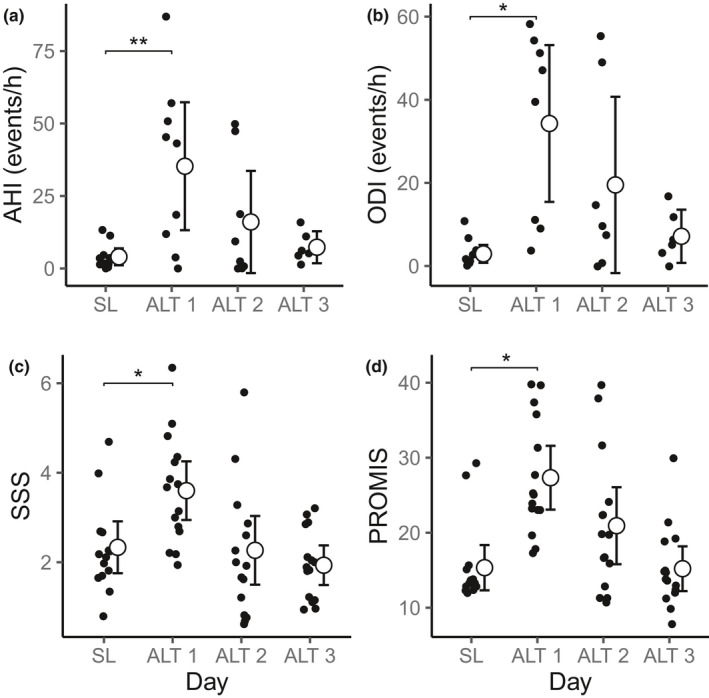
Sleep quality measures at sea level (SL) and over 3 nights at high altitude (ALT). Asterisks indicate significant differences from SL at *p *< 0.05 (*) or *p *< 0.01 (**) after correcting for multiple comparisons

Subjective sleep quality and SDB were worst on the first night at high altitude. Table 4 provides average sleep measures on each day. AHI increased significantly on night 1 at high altitude (Figure 2a), particularly due to an increase in hypopneas and central apneas and returned to baseline levels by day 3. The ODI demonstrated a similar pattern, increasing significantly on night 1 at high altitude (Figure 2b) but returned to baseline levels in most participants by night 2. While acute desaturation events improved by day 2, the mean and nadir sleep saturation remained significantly lower than baseline levels throughout the stay at high altitude. Wake after sleep onset (WASO) was longer at high altitude, although not significant after correcting for multiple comparisons, but returned to near baseline levels by night 3. The average sleep efficiency decreased at high altitude compared to sea level, but this change was also not significant after correcting for multiple comparisons. Subjective sleep quality measures were also impacted by the high altitude. SSS and PROMIS scores increased after the first night at high altitude but returned to baseline levels after the second night (Figure 2c,d).

**TABLE 4:** Effects of high altitude on SDB and subjective sleep quality measures

Variable	SL	ALT 1	*p*	ALT 2	*p*	ALT 3	*p*
AHI	4.3 (4.5)	35.3 (28.7)	0.014*	16.0 (21.1)	0.205	7.3 (5.3)	0.219
Hypopnea Index	2.8 (2.3)	20.9 (16.3)	0.014*	8.4 (16.0)	0.205	6.5 (4.9)	0.063
Apnea Index	0.5 (1.1)	0.3 (0.8)	0.423	0.1 (0.2)	1	0.1 (0.2)	1
Central Apnea Index	0.5 (0.6)	14.0 (17.1)	0.052	7.6 (15.2)	0.462	0.8 (0.8)	0.786
ODI	3.1 (3.3)	34.3 (22.6)	0.014*	19.5 (22.9)	0.078	7.2 (6.1)	0.063
Mean night‐time SpO_2_	94.7 (0.9)	77.0 (2.4)	0.014*	77.6 (2.9)	0.016	78.5 (1.6)	0.036
Nadir SpO_2_	85.8 (4.4)	65.3 (6.2)	0.014*	68.0 (6.4)	0.022	70.7 (3.6)	0.031
WASO	35.1 (19.9)	57.8 (35.9)	0.102	76.0 (52.4)	0.01	43.9 (37.6)	0.45
Sleep efficiency	83.8 (7.1)	79.7 (11.8)	0.123	72.6 (13.6)	0.005	77.5 (14.2)	0.52
SSS	2.3 (1.0)	3.6 (1.2)	0.017*	2.3 (1.4)	0.548	1.9 (0.8)	0.356
PROMIS T‐score	15.3 (5.5)	27.3 (7.7)	0.002*	20.9 (9.3)	0.053	15.2 (5.4)	0.548

Data are presented as mean (standard deviation). Raw *p* values are provided and asterisks indicate significant differences from SL after B‐H correction for multiple comparisons. Units: AHI (events/hour), hypopnea index (events/hour), apnea index (events/hour), central apnea index (events/hour), ODI (events/hour), SpO_2_ (%), WASO (min), sleep efficiency (%).

### 3.4 Effects of physiology and sleep quality on cognitive performance

**FIGURE 3:**
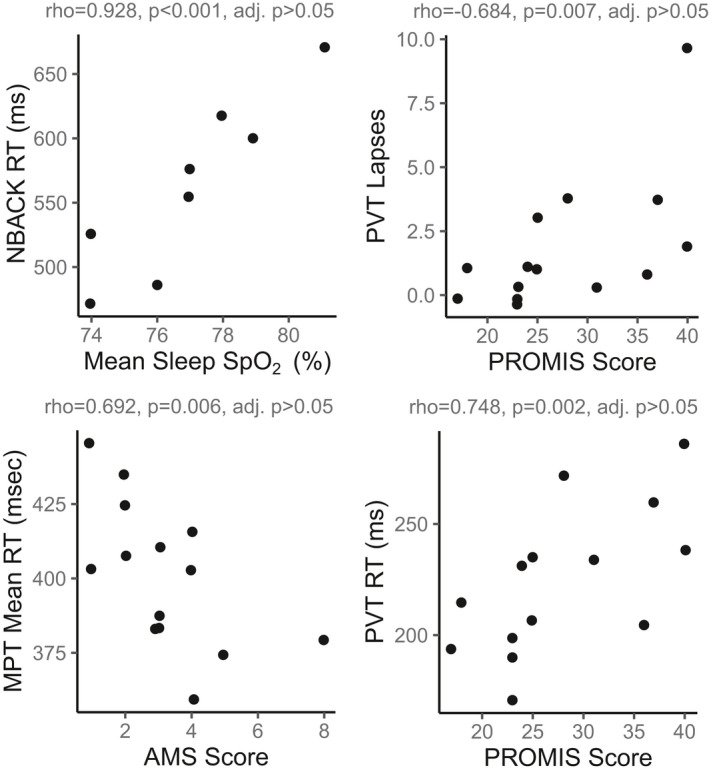
Cognition tests demonstrating significant associations with sleep quality measures and AMS scores after the first night at high altitude. Spearman's rho, raw p‐values, and p‐values after adjustment for multiple comparisons are provided

We aimed to determine if daytime hypoxemia, AMS, or SDB were the primary contributors to cognitive impairment at high altitude. Since sleep measures and AMS scores were worst on the first night at high altitude, we assessed associations between these variables and cognitive test performance in the morning following the first night at high altitude (ALT 1). We found scores on multiple cognitive function tests were associated with measures of sleep quality but not resting daytime SpO_2_. The top four significant correlations before correcting for multiple comparisons are provided in Figure 3 and an extended table with all comparisons is provided in Table S1.

Higher PROMIS scores (worse self‐reported sleep quality) were associated with slower reaction times and more lapses on the PVT (Figure 3). Similarly, individuals with the lowest sleep efficiency had slower reaction times on the NBACK (ρ = −0.648, *p* = 0.049) and VOLT (ρ = 0.685, *p* = 0.035) tests on average. Higher AMS scores were associated only with lower scores on the MPT (Figure 3). Unexpectedly, individuals with higher AHI and ODI scores performed better on the VOLT task (AHI: ρ = 0.798, *p* = 0.01; ODI: ρ = 759, *p* = 0.029). While some of these associations appear promising, due to a large number of tests and limited sample size, we were underpowered to detect these associations after correcting for multiple comparisons. Therefore, these relationships warrant further investigation.

## 4 DISCUSSION

### 4.1 Sleep during acclimatization

We found that subjective sleep quality was poor and SDB was common in unacclimatized sojourners to high altitude but improved to near baseline levels after 3 days of partial acclimatization at an altitude of 3800 m. Periodic breathing, the number of desaturation events, and subjective sleep quality worsened upon arrival to high altitude but improved to near baseline levels after 3 days of partial acclimatization. The AHI and ODI increases initially, primarily due to increased hypopnea and central apnea events, but returned to baseline levels by night 3. Despite these improvements, average sleep SpO_2_ remained low. However, the decrease in hypopneas and central apneas resulted in a slight increase in nadir SpO_2_ from night 1 to night 3. Overall sleep efficiency was also poorer on average each night at high altitude and was the lowest on night 2 when WASO was also the longest. These objective measures were reflected in increased subjective SSS and PROMIS T‐scores on the morning following the first night at high altitude.

Our results support the consensus that sojourners to high‐altitude experience poor‐quality sleep linked to periodic breathing (Ainslie et al., 2013; Weil, 2004). Whether or not acclimatization improves or worsens SDB may depend on the severity of the altitude exposure, as well as individual variation in peripheral and central ventilatory chemoreflex sensitivity. One hypothesis is that periodic breathing should worsen with acclimatization as the hypoxic ventilatory response increases over time (Ainslie et al., 2013; Hupperets et al., 2004; Pamenter & Powell, 2016), leading to high loop gain and breathing instability manifesting as more vigorous and/or frequent bouts of hyperventilation and subsequent apneas. However, our data and that of others (Tseng et al., 2015; Weil, 2004; White et al., 1987; Wickramasinghe & Anholm, 1999) indicate periodic breathing and overall sleep quality (measured objectively or subjectively) improves by day 3 at an elevation of 3800 m. In comparison, studies in acclimatized sojourners at very high altitude find persistent periodic breathing and sleep disturbance even after acclimatization (Bloch et al., 2010; Nussbaumer‐Ochsner et al., 2012). This finding indicates a potential interaction between elevation and acclimatization‐induced changes in ventilatory control on periodic breathing and sleep disturbance.

At high altitudes, periodic breathing may manifest in unacclimatized sojourners as a result of acute hypoxic ventilatory response‐induced hyperventilation, which reduces arterial PCO_2_ below the ventilatory recruitment threshold and generates an apnea until PCO_2_ recovers. However, as the ventilatory recruitment threshold decreases with acclimatization (Slessarev et al., 2010), this instability may be ameliorated. However, at higher altitudes, the gain of the hypoxic ventilatory drive becomes increasingly higher and would exacerbate breathing instability and SDB (Orr et al., 2017).

### 4.2 Cognitive function during acclimatization

The cognitive function test displaying the largest, and most consistent, impairment at high altitude was the PVT. This test measures sustained attention and reaction times to a visual stimulus. We found PVT reaction times were higher on the first day at high altitude and remained significantly higher on day 3 despite AMS, AHI, and ODI scores returning to baseline levels. These results are consistent with Pun et al., (2018) who found significant increases in PVT reaction time after 1 day at high altitude and significant improvement by day 6. While we do see an improvement in average PVT from day 2 to 3 at high altitude, full recovery of PVT performance may take longer than 3 days. Furthermore, Pun et al., (2018) found a significant correlation between PVT reaction time and AMS scores calculated by the Environmental Symptom Questionnaire – Cerebral (Sampson et al., 1983). We did not find such an association with AMS score calculated via Lake Louise Score. However, we did find that poor sleep measured subjectively by the PROMIS and SSS may be associated with lower PVT performance. The PVT is particularly resistant to learning effects and, whereas the variation in response time may decrease with repeated administration in the same individuals, the mean reaction time is not impacted (Basner et al., 2018).

The mean reaction time on the BART improved with each administration of the task, despite no change in the total number of pumps per balloon. Pighin et al., (2020) used the same test in subjects exposed acutely to normobaric hypoxia with 7 days between testing periods. They found that individuals used a higher number of pumps before collecting their reward in hypoxia versus normoxic control tests, suggesting increased risk‐taking behavior in hypoxia. While we did not find differences in the total number of pumps per balloon, the faster reaction time may also be an indicator of increased risk‐taking behavior as there is less time for consideration between each action. Alternatively, this result may be attributed to learning effects as participants become more comfortable with the number of pumps they can attempt per balloon despite the test being designed to discourage learning by having each balloon pop after a random number of pumps. This learning effect is supported by the fact that individuals who completed their sea‐level cognition testing last had significantly faster reaction times on the BART than individuals who completed their sea‐level testing first (first: 608 ± 238; last: 340 ± 145 ms, *p* = 0.026). Therefore, while there may be important changes in risk‐taking behavior at high altitude, improved methods for measuring this trait in the same individuals over subsequent days may be necessary to determine how these effects change with acclimatization.

### 4.3 Is sleep a key determinant of cognitive performance at altitude?

Of note, there was no association between resting daytime SpO_2_ and performance on any cognitive function task. Instead, these results suggest that poor sleep quality and SDB contributed to daytime impairments in sustained attention and reaction times on the PVT in unacclimatized sojourners to high altitude. While acute hypoxia exposure experiments demonstrate hypoxemia itself can produce cognitive impairment (Malle et al., 2016; Phillips et al., 2015), it seems that overnight partial acclimatization may play a role in mitigating these effects and that the effects of poor sleep at high altitude on daytime cognition cannot be discounted.

To our knowledge, this is the first study to measure the impact of acclimatization to high altitude on comprehensive neurocognitive performance coupled with actigraphic and polygraphic measures of SDB. Nonetheless, the study has some important limitations. Our sample size was relatively small, largely due to the housing capacity of the high‐altitude facility, equipment limitations, and participant adherence to Actiwatch and respiratory polygraphy procedures. We may have also been underpowered to detect associations between cognitive performance and physiological and sleep variables, particularly after correcting for multiple comparisons. With 16 cognitive function variables and 13 physiology and sleep variables, a total of 208 correlations were examined. We also note the potential for learning effects on the neurocognitive testing, which would artificially increase the apparent impact of acclimatization on improvement. To mitigate learning effects, we assigned half of the participants to complete sea‐level testing before ascent, and a half after returning to sea level. In addition, the *Cognition* test battery allows for within‐subject repeated testing by producing novel permutations of each test in each session. To control for learning completely, a much more logistically complex study design would be needed and may limit feasibility. We note that learning effects on this neurocognitive battery are low, with the potential exception of the BART. The PVT is particularly resistant to learning as it is based only on attention and reaction time.

In conclusion, our data show that SDB is common in unacclimated sojourners to moderately high altitude, but that subjective sleep quality and SDB return to sea‐level values after 3 nights of partial acclimatization. Furthermore, of the cognitive domains tested, it appears that PVT performance is most influenced by high‐altitude exposure and that poor sleep may impair sustained attention at high altitude. Additional research examining how sleep quality contributes to daytime cognitive performance at high altitude is warranted. Such future studies should implement full polysomnography since variation in sleep stage durations during acclimatization may also impact daytime cognition. Furthermore, future studies may consider the use of hypobaric chambers for simulating high altitude since sleep quality may be impacted by the transition to a new environment in field studies, and chamber experiments would allow researchers to account for this effect with blinded placebo control.^1^

## AUTHORS' CONTRIBUTIONS

ECH, JEO, RB, PD, and AM designed and conceived the research. Experiments were performed by ECH, RB, KP, NP, BO, and SF. JEO, PD, and RB analyzed the raw polysomnography data and the final data analysis was conducted by SJ, SS, JEO, and ECH. The manuscript was drafted by SF, BO, JEO, and ECH, then edited and approved by all authors. ResMed provided a philanthropic donation to UC San Diego. AM reports income related to medical education from Equillium, Corvus, and Livanova.

## Supporting information



Supplement S1

Supplement S2

## Footnotes

^1^ At the request of the authors, readers are herein alerted to the fact that additional materials related to this manuscript may be found at https://doi.org/10.6086/D1Z404. These materials are not a part of this manuscript and have not undergone peer review by the American Physiological Society (APS). APS and the journal editors take no responsibility for these materials, for the website address, or for any links to or from it.
