# Supplementary material for: Improvements in sleep‐disordered breathing during acclimatization to 3800 m and the impact on cognitive function
Source: Physiol Rep. 2021 May 15;9(9):e14827. doi: 10.14814/phy2.14827 (PMC8123551; doi:10.14814/phy2.14827)
Supplement: Supplementary file 1 — Supplement S1 [file PHY2-9-e14827-s001.pdf]

Supplemental Table 1. Spearman's correlations for physiology and sleep data associations with cognitive test performance.

Test	Measure	Day SpO_2	AMS	AHI	*Hypop. Index*	*Apnea Index*	*Central Apnea Index*	ODI	Avg. Night SpO_2	*T80*	Sleep Effic.	WASO	PROMIS	SSS
PVT	Mean reaction time	-0.119 (0.684)	0.126 (0.668)	0.433 (0.25)	0.367 (0.336)	0.251 (0.515)	0.525 (0.146)	0.524 (0.197)	0.566 (0.143)	-0.333 (0.428)	-0.358 (0.313)	0.079 (0.838)	**0.748 (0.002)**	-0.027 (0.926)
	Lapses	-0.208 (0.475)	0.276 (0.34)	0.436 (0.241)	0.333 (0.381)	0.316 (0.407)	0.513 (0.158)	0.577 (0.134)	0.553 (0.155)	-0.319 (0.441)	-0.499 (0.142)	0.075 (0.837)	**0.684 (0.007)**	-0.028 (0.924)
	False starts	-0.22 (0.45)	0.147 (0.617)	-0.017 (0.965)	-0.373 (0.323)	-0.356 (0.347)	0.353 (0.352)	-0.704 (0.051)	-0.613 (0.106)	0.704 (0.051)	0.131 (0.719)	0.449 (0.193)	-0.253 (0.383)	-0.448 (0.108)
BART	Mean reaction time	0.084 (0.775)	-0.716 (0.004)	-0.3 (0.437)	-0.5 (0.178)	**-0.707 (0.033)**	0.186 (0.631)	-0.571 (0.151)	-0.675 (0.066)	**0.881 (0.007)**	0.224 (0.537)	-0.37 (0.296)	-0.257 (0.376)	-0.041 (0.89)
	Pumps	0.349 (0.221)	0.126 (0.668)	-0.267 (0.493)	0.05 (0.912)	0.114 (0.77)	-0.576 (0.104)	0.214 (0.619)	0.265 (0.526)	-0.619 (0.115)	0.2 (0.584)	-0.2 (0.584)	-0.166 (0.571)	0.313 (0.275)
DSST	Mean reaction time	-0.44 (0.116)	0.171 (0.56)	-0.2 (0.613)	-0.267 (0.493)	-0.023 (0.954)	0.153 (0.695)	-0.238 (0.582)	-0.133 (0.754)	0.476 (0.243)	-0.394 (0.263)	-0.43 (0.218)	0.363 (0.202)	-0.186 (0.524)
	Correct responses	0.42 (0.135)	-0.169 (0.564)	0.151 (0.698)	0.21 (0.587)	-0.023 (0.953)	-0.171 (0.66)	0.193 (0.647)	0.03 (0.943)	-0.386 (0.346)	0.426 (0.22)	0.383 (0.275)	-0.397 (0.159)	0.145 (0.622)
LOT	Mean reaction time	0.477 (0.084)	-0.512 (0.061)	0.45 (0.23)	0.517 (0.162)	0.183 (0.638)	0.136 (0.728)	0.452 (0.267)	0.265 (0.526)	-0.048 (0.935)	-0.03 (0.946)	-0.164 (0.657)	-0.305 (0.288)	-0.204 (0.483)
	Correct responses	0.323 (0.261)	0.253 (0.383)	0.559 (0.117)	**0.695 (0.038)**	**0.743 (0.022)**	0.069 (0.86)	0.317 (0.444)	**0.753 (0.031)**	-0.586 (0.127)	-0.325 (0.359)	0.117 (0.748)	-0.21 (0.47)	0.005 (0.988)
NBACK	Mean reaction time	0.29 (0.315)	0.314 (0.274)	0.317 (0.41)	0.3 (0.437)	0.548 (0.127)	-0.237 (0.539)	0.69 (0.069)	**0.928 (<0.001)**	**-0.833 (0.015)**	**-0.648 (0.049)**	-0.103 (0.785)	0.442 (0.113)	0.466 (0.093)
	Correct responses	0.055 (0.853)	-0.347 (0.224)	0.209 (0.589)	0.31 (0.417)	0.16 (0.68)	-0.204 (0.598)	0.515 (0.192)	0.467 (0.244)	-0.467 (0.243)	-0.11 (0.763)	-0.5 (0.141)	0.127 (0.665)	0.247 (0.394)
VOLT	Mean reaction time	-0.033 (0.91)	0.054 (0.855)	-0.083 (0.843)	-0.417 (0.27)	-0.023 (0.954)	-0.051 (0.897)	-0.238 (0.582)	0.229 (0.586)	0.024 (0.977)	**-0.685 (0.035)**	-0.115 (0.759)	0.137 (0.64)	0.459 (0.099)
	Correct responses	0.327 (0.253)	0.053 (0.856)	**0.798 (0.01)**	**0.714 (0.031)**	**0.713 (0.031)**	0.299 (0.434)	**0.759 (0.029)**	**0.848 (0.008)**	**-0.892 (0.003)**	-0.372 (0.29)	0.354 (0.316)	0.253 (0.383)	0.343 (0.229)
AM	Mean reaction time	0.261 (0.368)	0.213 (0.464)	-0.3 (0.437)	-0.317 (0.41)	-0.16 (0.681)	-0.153 (0.695)	0 (>0.99)	0.012 (0.977)	-0.286 (0.501)	-0.018 (0.973)	0.345 (0.331)	-0.144 (0.624)	0.182 (0.534)
	Correct responses	-0.125 (0.67)	-0.306 (0.287)	-0.393 (0.295)	-0.536 (0.137)	-0.527 (0.145)	-0.136 (0.727)	-0.357 (0.389)	-0.337 (0.414)	0.286 (0.501)	0.146 (0.687)	-0.122 (0.737)	0.013 (0.964)	0.122 (0.678)
MPT	Mean reaction time	0.04 (0.893)	**-0.692 (0.006)**	-0.183 (0.644)	-0.4 (0.291)	**-0.707 (0.033)**	0.17 (0.663)	-0.19 (0.665)	-0.386 (0.346)	0.524 (0.197)	-0.103 (0.785)	-0.224 (0.537)	0.23 (0.429)	0.329 (0.25)

Values are provided as Spearman's rho (p-value). No correlations were significant after correcting for multiple comparisons (adj. $p > 0.05$ for all).
