# Supplementary material for: Improvements in sleep‐disordered breathing during acclimatization to 3800 m and the impact on cognitive function
Source: Physiol Rep. 2021 May 15;9(9):e14827. doi: 10.14814/phy2.14827 (PMC8123551; doi:10.14814/phy2.14827)
Supplement: Supplementary file 2 — Supplement S2 [file PHY2-9-e14827-s002.pdf]

Detailed descriptions of tests in the *Cognition* test battery:

Psychomotor vigilance task (PVT) tests vigilant attention by recruiting the prefrontal, motor, and visual cortices. This test features a black screen with one centered rectangle outlined in red. At random intervals, numbers (a timer) will appear inside the rectangle. The participant's goal is to respond and click the screen as fast as possible when the timer appears. However, if a user clicks before the timer appears it counts as a false start.

Balloon analog risk task (BART) is a measure of the participants risk decision making and uses various parts of the brain including the orbital frontal cortex, amygdala, hippocampus, and the anterior cingulate cortex. In this test the user will see a total of 30 balloons. The user is awarded $1.00 per pump of air into the balloon and given the option to inflate a balloon or collect the winnings. The user is warned that each balloon may pop at random. If the balloon pops the rewards gained for the balloon cannot be collected. The aim is to collect the highest earnings in the shortest amount of time.

Digit symbol substitution task (DSST) tests complex scanning, mental flexibility, and visual tracking abilities. This test recruits the temporal, prefrontal, and motor cortices. The middle of the screen has a blue box that will display a symbol. Displayed along the bottom of the screen is a list of numbers along with corresponding symbols. The goal is to correctly hit the number which corresponds with the displayed symbol.

Line orientation task (LOT) is a test of spatial orientation, using the right temporo-parietal and visual cortices. Two lines appear on the screen, one is black and one is blue with a circle in the middle of it. The black line is fixed and cannot be moved. The goal is to rotate the blue line so that it is parallel to the black line. This can be achieved by hitting a left arrow to rotate counterclockwise or a right arrow which rotates the line clockwise. The lines can vary in length and location.

NBack is a test of working memory which recruits functions from the dorsolateral prefrontal cortex, cingulate, and the hippocampus. In this test a series a differently colored fractal-like images are shown. The goal is to tap the screen when the current image matches the image shown two screens ago.

Visual object learning task (VOLT) assesses visual learning and spatial working memory using the medial temporal cortex. This task starts by showing the participant a series of 10 3-dimensional shapes. These shapes can be of different sizes and shading. The user is then shown a set of 20 shapes and asked to rank their remembrance of the shapes with varying degrees of certainty. The answers are either "definitely yes" or "probably yes" that they have seen this shape before, or "probably no" or "definitely no" that they have not seen this shape before.

Abstract matching (AM) measures abstraction ability testing the prefrontal cortex. Users are shown a shape and asked to pick the pair of shapes that fits best with that single shape. This can include different sets of shapes, colors, shading, and lining.

Motor praxis task (MPT) tests sensory motor speed using the sensorimotor cortex. In this test, boxes appear one at a time on the screen. The user must tap the box to make is disappear. The boxes get progressively smaller as more boxes are tapped.

More information and video demonstrations can be found at:
https://admin.joggleresearch.com/Home/Tasks
